# Core domain mutant Y220C of p53 protein has a key role in copper homeostasis in case of free fatty acids overload

**DOI:** 10.1007/s10534-015-9886-0

**Published:** 2015-10-05

**Authors:** Mario Arciello, Alessia Longo, Carmela Viscomi, Concetta Capo, Antonio Angeloni, Luisa Rossi, Clara Balsano

**Affiliations:** Department of Internal Medicine and Medical Specialties, “Sapienza” University of Rome, viale del Policlinico 155, 00161 Rome, Italy; Laboratory of Molecular Virology and Oncology, Francesco Balsano Foundation, Rome, Italy; Department of Biology, University of Rome “Tor Vergata”, Rome, Italy

**Keywords:** P53, Copper, Metal homeostasis, Fatty acid, Hepatocyte

## Abstract

Nonalcoholic fatty liver disease (NAFLD) is a pathology that includes a wide variety of clinical conditions ranging from simple steatosis to end-stage liver diseases. Despite the huge amount of researches, the molecular basis of NAFLD are still not fully understood. Recently, it was suggested a role for p53 in NAFLD pathogenesis. Among its targets there is Synthesis of Cytochrome c Oxidase 2 (SCO2), a copper chaperone, involved in both aerobic respiration and metal cellular excretion. Copper seems to play a role in NAFLD. It was demonstrated a low hepatic copper content in NAFLD patients, which correlates with metabolic syndrome parameters. Copper homeostasis deregulation, in fact, seems to be related to lipid metabolism alteration and insulin resistance. Here we provide evidence on the role of p53 in the modulation of copper homeostasis, in an experimental model of NAFLD. We used two different hepatoma cell lines, HepG2 and Huh 7.5.1, characterized by the presence of wt p53 and its Y220C mutant, respectively, treated with a free fatty acids (FFAs) solution. Interestingly, p53 activation correlated with the intracellular copper level maintenance. We demonstrated that, in hepatoma cell lines, core domain mutant Y220C of p53 affects the modulation of SCO2 and Copper transporter 1 (CTR1), influencing, in this way, intracellular copper homeostasis in presence of FFAs accumulation, and that the 220 residue of the protein is crucial for such control. The role of p53 we highlighted may have deep implications in clinical conditions where copper homeostasis is deregulated.

## Introduction

Nonalcoholic fatty liver disease (NAFLD) is the most diffuse chronic liver disease in developed countries, where its prevalence ranges from 10 to 30 %. NAFLD is frequently associated with the metabolic syndrome (MeS), of which it is considered the hepatic manifestation (Vanni et al. 2010; Veteläinen et al. 2007), as well as a pathogenic factor for its occurrence (Anstee et al. 2013). This pathology refers to a wide variety of clinical conditions ranging from simple steatosis to the non-alcoholic steatohepatitis (NASH). The latter, in particular, might further progress to end-stage liver diseases until to hepatocarcinoma (HCC) (Schreuder et al. 2008; Starley et al. 2010).

In the last decade, the number of proteins involved in the modulation of metabolism has been gradually increased. Among them, p53 protein, primarily known as a tumor suppressor, has been proposed as a new player in NAFLD pathogenesis, and growing evidences highlight its relevance as metabolic modulator (Panasiuk et al. 2006; Derdak et al. 2013).

The protein is encoded by the TP53 gene, located on the human chromosome 17 (17p13). It is a transcription factor (phospho-protein) that is found mutated in more than 50 % of all human tumors, including HCC. Among the several known p53 somatic missense mutations, of interest is the Y220C, which represents 1.4 % of them (Petitjean et al. 2007; Joerger and Fersht 2008). It is characterized by a tyrosine substitution with a cysteine in its core domain, leading to a reduced thermodynamic stability of the protein (Joerger et al. 2006) and to a different lipid metabolism modulation (Gori et al. 2014).

p53 modulates the activity of fructose-2,6-bisphosophatase, known as TIGAR (TP53-induced glycolysis regulator), and SCO2 (Synthesis of Cytochrome c Oxidase 2) protein, influencing, in this way, cellular glycolysis and respiration, respectively (Zhang et al. 2010; Won et al. 2012).

SCO2, in particular, is a copper chaperone extremely important for cell respiration, because it is crucial for the proper assembly and function of the complex IV of the mitochondrial electron transport chain, Cytochrome c Oxidase (Cytox), and for the distribution of copper to the ATPases (ATP7A and B), responsible of the cell copper excretion (Leary et al. 2007). This latter ability could be of particular relevance and interest in NAFLD pathogenesis, because patients affected by this disease are characterized by low hepatic copper content (Aigner et al. 2008; Aigner et al. 2010; Medici 2013). Furthermore, it was observed that a reduced copper intake promotes the biosynthesis of fatty acids and cholesterol, increases lipoproteins levels, therefore worsens the progression of liver steatosis (Wilson et al. 1997).

Here, we investigated, in hepatoma cell lines, the involvement of p53, and of its core domain mutant Y220C, in the modulation of copper homeostasis in the presence of free fatty acids overload.

## Materials and methods

### Cell cultures and FFAs treatment

Two human hepatoma cell lines characterized by different forms of p53 were used: HepG2 cells which express wild-type (wt) p53, and Huh7.5.1 which express Y220C p53 mutant.

HepG2 and Huh 7.5.1 cells, purchased from American Type Culture Collection (ATCC), were maintained in culture at 37 °C in 5 % CO2 in Dulbecco’s modified Eagle’s medium (DMEM; Lonza East Rutherford, NJ, USA), containing 1 % l-glutamine, 10,000 U/mL of penicillin and streptomycin (Lonza) and 10 % fetal bovine serum (Gibco, Milan, Italy).

Long-chain FFAs, palmitic acid (PA; 16:0) and oleic acid (OA; 18:1) (Sigma-Aldrich, Milan, Italy) were dissolved in methanol (MetOH). Steatosis was induced as previously described by Ricchi et al. (2009). Briefly, cell cultures were incubated with: DMEM containing 10 % of Charcoal stripped fetal bovine serum (FBS Charcoal; Lonza), 1 % bovine serum albumin (BSA), and 1 % l-glutammine supplemented with a solution of FFAs, PA (0.16 mM) and OA (0.33 mM) in a 1:2 molar ratio at a final concentration of 0.5 mM for 14 and 24 h. Cell cultures were incubated with MetOH were considered as control.

### Intracellular lipid content evaluation by AdipoRed assay

The intracellular increase of lipid content was evaluated by AdipoRed assay (Lonza); according to the manufacturer’s protocol. This assay exploits the properties of the dye Nile Red that binds lipids and becomes fluorescent when it is in a hydrophobic environment. Cells were grown in 96 blackplates and treated with FFAs. At the end of incubation cells were washed with phosphate buffered saline (PBS) and incubated with AdipoRed for 10 min. After incubation, fluorescence was evaluated and expressed as relative fluorescence units (RFLU) per mg of protein and depicted as fold of increase vs control (vehicle-treated cells).

### Cell viability and cytotoxicity determination

Cytotoxicity of the FFAs treatment and cell viability were assessed using the MTS Cell proliferation assay (Promega, WI, USA), according to the manufacturer’s protocol, and the Trypan Blue exclusion test.

### RNA extraction and quantitative real-time polymerase chain reaction (RT-PCR) analysis

Total RNA was isolated using the Trizol reagent (Life Technologies, Carlsbad, CA, USA) and cDNA was prepared using Reverse Transcription System (A3500, Promega), according to the manufacturer’s protocol. Human PCR primers were designed IDT Integrated DNA Technologies and all purchased from BIO-FAB research (Rome, Italy). Primers used were the following: p53, 5′-AGAGCTGAATGAGGCCTTGGAACT-3′ and 5′-GGCCCTTCTGTCTTGAACATGAGT-3′; p21, 5′-TCACACCATGACAAGACTCTC-3′ and 5′-AAATGCCAGTCACTTAGTACAG-3′; SIRT1, 5′-CAGGTTGCGGGAATCCAAAGGATA-3′ and 5′-TCCTCGTACAGCTTCACAGTCAAC-3′; CTR1, 5′-CGTAAGTCACAAGTCAGCATTCGC-3′ and 5′-AGGTACCCGTTGTAGGTCATGAAG-3′; SCO2, 5′-TCCATTGCCATCTACCTGCTCAAC-3′ and 5′-TCAAGACAGGACACTGCGGAA-3′; ATP7B, 5′-CTCATTAAAGCTACCCACG-3′ and 5′-GACAAAATATCCACTAAACCG-3′; ATP7A, 5′-GACCCTACAGGAAGCTATT and GCCGTAACAGTCAGAAAC. Primers for β-actin were used as housekeeping controls: β-actin, 5′-GCACTCTTCCAGCCTTCC-3′ and 5′-AGGTCTTTGCGGATGTCCAC-3′. RT-PCR was performed with the 7500 Fast Real-Time PCR System (Applied Biosystems, Life Technology) using SYBR Green as fluorescence dye.

Gene expression profiling was performed using the comparative cycle threshold (Ct) method of relative quantification (DDCt; the reference sample was the control) through the instrument’s software (7500 Software v2.0.5, Applied Biosystems). Data are expressed as log2 of the relative quantification (RQ) defined also as fold changes versus control.

### Immunoblot analysis

Cell lysates were analysed in denaturing conditions through SDS-PAGE. Primary antibodies were as follows: rabbit polyclonal anti-p53 (FL-393), rabbit polyclonal anti-SIRT1 (H-300), goat policlonal anti-CTR1 (G-15), rabbit polyclonal anti-ATP7B (H-94), goat polyclonal anti-Actin (I-19) (Santa Cruz Biotechnology, CA, USA); rabbit polyclonal anti-phospho-p53 (Ser 15) (#9284), rabbit polyclonal acetyl-p53 (Lys382) (#2570) (Cell Signaling Technology, Inc., Merck-Millipore, MA, USA), rabbit policlonal anti-SCO2 (ab58814), mouse monoclonal anti-ATP7A (S60-4) (ab131400) (all from Abcam, UK). Antibody detection was performed by Amersham Hyperfilm ECL (GE Healthcare Life Sciences, USA). Densitometric analysis of immunoblots was performed by ImageJ64 image processing software for electrophoresis gel analysis.

### Determination of intracellular copper content

Before analysis, cells in hypotonic PBS were lysated by sonication (for 20 s) and then diluted 1:2 (v:v) with 65 % nitric acid. After at least one week at room temperature, copper content was assayed by atomic absorption spectroscopy using an AAnalyst 300 instrument equipped with a graphite furnace with platform (HGA800) and an AS-72 autosampler (Perkin-Elmer, Waltham, MA, USA).

### Cytochrome c oxidase assay

Cytochrome *c* oxidase (Cytox) was assayed spectrophotometrically in 30 mmol/L phosphate buffer, pH 7.4, 25 °C by following the oxidation of reduced cytochrome c (from horse heart; Sigma) (0.02 mmol/L) at 550 nm. A Beckman-Coulter DU800 spectrophotometer (Beckman-Coulter, Fullerton, CA, USA) was used. Activity was expressed as relative variation evaluated versus control cells.

### Transfections and establishment of stable cell lines

Huh7.5.1 cells were plated in antibiotic-free cell growth medium, and after 24 h we performed transfection using Lipofectamine 2000 Transfection Reagent and Opti-MEM I Reduced Serum Medium (Life Technologies), according to manufacturer’s protocol. Wild-type TP53 gene was cloned into pcDNA3-HA expression vector (Addgene, USA), and 10 µg were transfected. The same amount of the pcDNA3-HA empty vector were transfected as negative control. After 24 h fresh medium containing neomycin (G418, G8168 Sigma-Aldrich) was added daily to select stable clones.

### Statistical analysis

Results are expressed as mean ± standard deviation (SD). All experiments were performed at least in triplicate. Statistical analysis were performed by Student’s *t* test for unpaired data and the differences were considered statistically significant at *p < 0.05; **p < 0.01; ***p < 0.001.

## Results

### FFAs treatment induces a different intracellular lipid content increase in HepG2 and Huh 7.5.1 cell lines

To set-up an in vitro model of steatosis we used two hepatoma cell lines, HepG2 and Huh 7.5.1, characterized by the presence of the wild-type (wt) and the core mutant (Y220C) form of the p53 protein, respectively. Cell lines were treated with a solution containing oleic and palmitic acids in a molar ratio of 2:1. We used these fatty acids in order to mimic the Western diet, and because they belong to serum tryglicerides, in both healthy and NAFLD patients (Baylin et al. 2002; Gómez-Lechón et al. 2007).

**Fig. 1:**
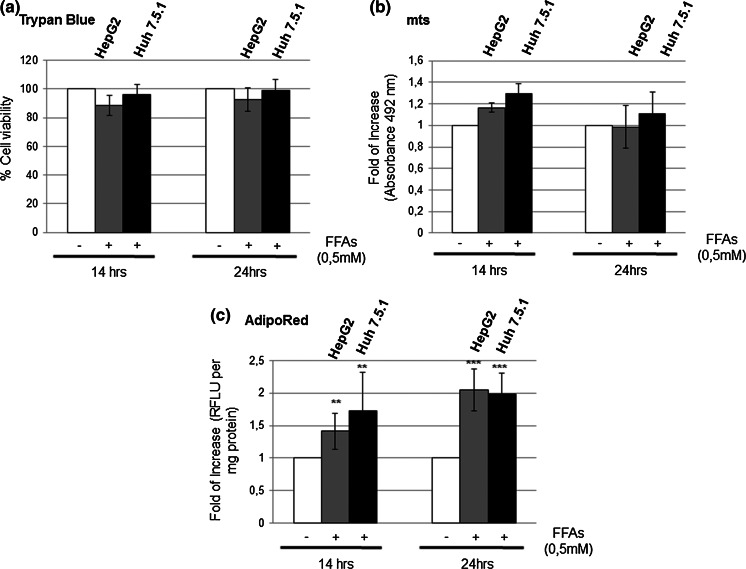
Fatty acids treatment for 14 and 24 h causes an intracellular lipid accumulation in both HepG2 and Huh7.5.1 cells without affecting cell viability. **a** After 14 and 24 h incubation with FFAs, cells were detached and observed under a light microscope in the presence of the vital stain Trypan Blue. Cells excluding the stain were considered viable. We reported the percentage of viable cells compared to controls (100 %) obtained in at least four experiments ±SD. *p < 0.05; **p < 0.01; ***p < 0.001. **b** Evaluation of FFAs treatment cytotoxicity through the MTS assay in HepG2 and Huh7.5.1 cells. Control cells were referred to as 1 in the graph. All *data* are expressed as mean ± SD of at least four independent experiments. *p < 0.05; **p < 0.01; ***p < 0.001. **c** Adipored assay of cells treated for 14 and 24 h with FFAs to evaluate intracellular lipid accumulation. *Data* are presented as the mean of the fold of increase of Relative Fluorescence Units (RFLU) per mg of protein ±SD compared to controls. *Data* reported were obtained by at least four experiments. *p < 0.05; **p < 0.01; ***p < 0.001

In order to avoid cell toxicity and to obtain a significant intracellular lipid accumulation, we performed a FFAs preliminary dosage set-up, from 0.1 up to 2 mM (data not shown). Finally, we chose the final concentration of 0.5 mM to treat cells for 14 and 24 h (Fig. 1a, b). The Adipored assay revealed a progressive intracellular lipid content rise, similar in both cell lines, which reached about 1,5 folds of increase after 14 h, and 2 folds after 24 h compared to untreated cells (Fig. 1c). Interestingly, HepG2 cells showed a slower and progressive increase of lipid intake with respect to Huh 7.5.1 (Fig. 1c).

### FFAs treatment influences the activity of wt p53 and its Y220C mutant differently

In a previous work we demonstrated, in HepG2 and Huh7.5.1, a different modulation of the fatty acids beta-oxidation pathway by p53, depending on the presence of wt p53 or the Y220C p53 mutant (Gori et al. 2014).

**Fig. 2:**
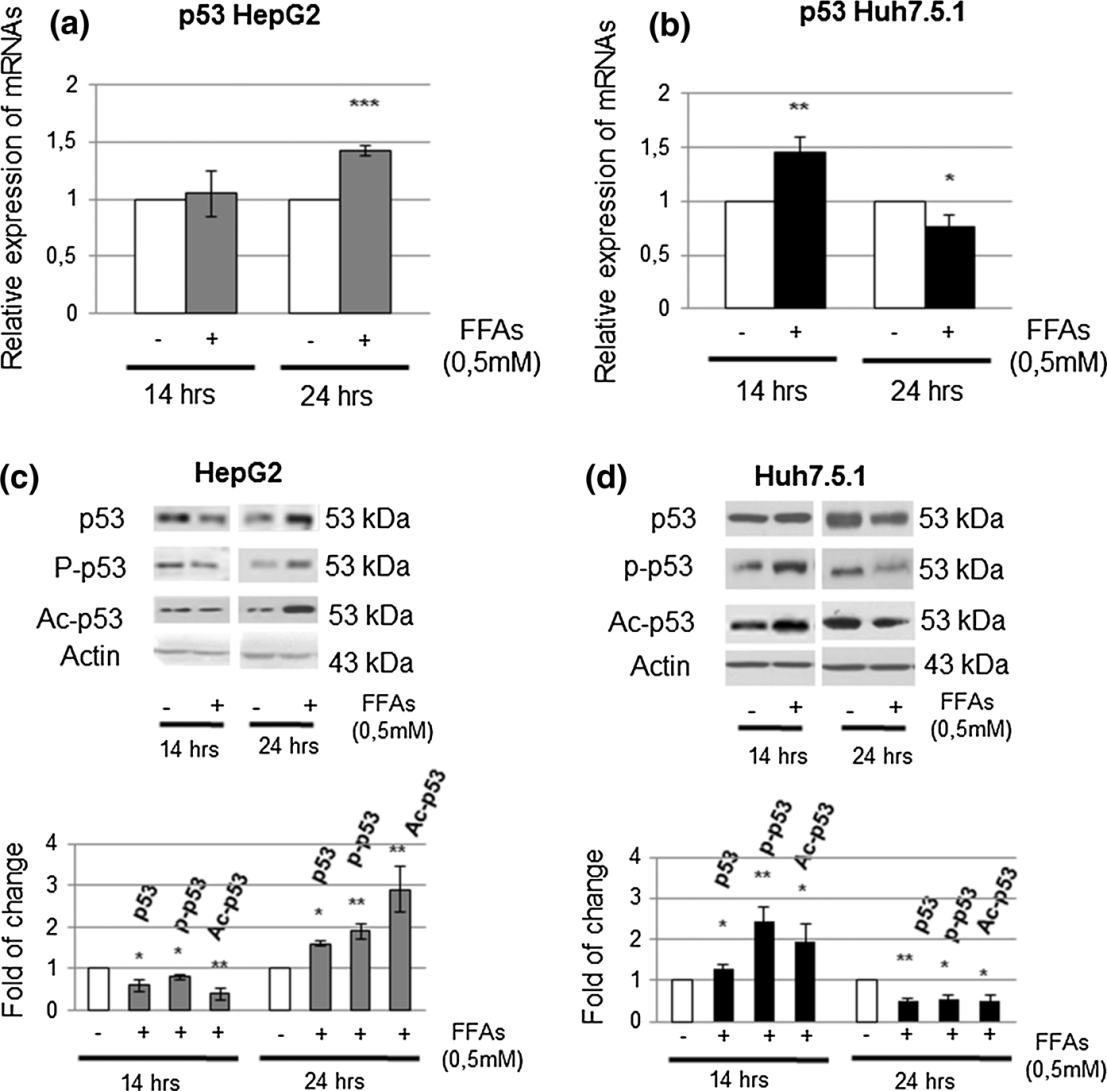
FFAs treatment provokes p53 response and activation in both HepG2 and Huh7.5.1 cells but with a different timing. **a** and **b** Relative expression of p53 mRNA, by real time-polymerase chain reaction (RT-PCR), in HepG2 and Huh 7.5.1 treated cells vs control (referred to as 1 in the graphs) respectively. β-actin was used as housekeeping gene. Data are representative of four independent experiments and are expressed as mean ± SD. *p < 0.05; **p < 0.01; ***p < 0.001. **c** and **d** Protein expression of p53, phospho-p53 (Ser15) and acetyl-p53 (Lys382) was evaluated in HepG2 and Huh 7.5.1 by Western Blotting with relative densitometric analysis, in which the expression level of each protein was normalized *vs* the corresponding β-actin, which was used as a loading control (*bottom panels*). *Data* showed are representative of at least three independent experiments and are expressed as fold of change compared to the respective controls ±SD. *p < 0.05; **p < 0.01; ***p < 0.001

Here, we highlighted a different timing in the induction of p53 transcription, in response to FFAs. In particular, RT-PCR experiments revealed that the transcript of the wt p53 increased after 24 h of FFAs treatment (Fig. 2a), while the Y220C p53 was already up-regulated at 14 h (Fig. 2b).

**Fig. 3:**
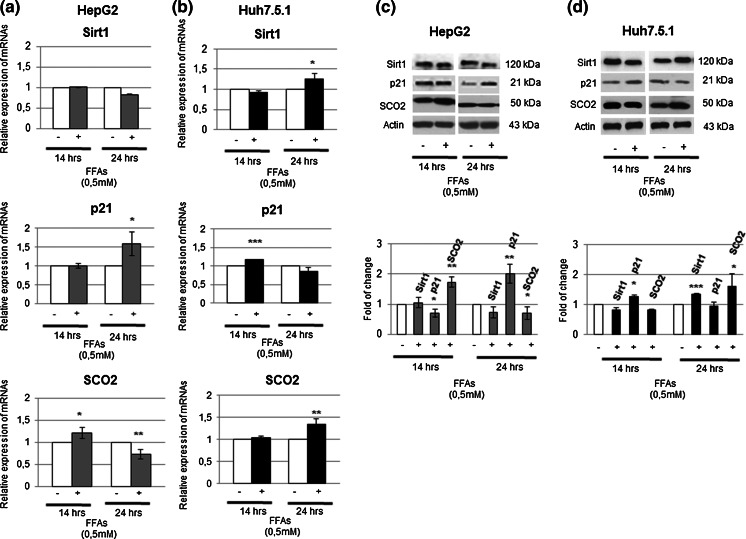
Molecular investigations of genes related to p53 activation after FFAs treatments for 14 and 24 h in both HepG2 and Huh7.5.1 cells. **a** and **b** Gene expression of Sirt1, p21 and SCO2 was evaluated by RT-PCR analysis in HepG2 and Huh 7.5.1 after 14 and 24 h; β-actin was used as housekeeping gene. Data are representative of four experiments and are expressed as mean ± SD. *p < 0.05; **p < 0.01; ***p < 0.001. **c** and **d** The corresponding protein expressions were evaluated by Western Blotting (with relative densitometric analysis) in HepG2 and HuH7.5.1; β-actin was used as a loading control. *Data* are representative of four independent experiments. *Data* shown are presented as fold change relative to the controls and are expressed as the mean ± SD. *p < 0.05; **p < 0.01; ***p < 0.001

When we looked at the activity of p53 protein, investigating its Ser 215 phosphorylation and Lys 382 acetylation status, we highlighted an earlier activation of the Y220C p53 protein with respect to wt p53 (Fig. 2c, d). To further confirm and investigate the effects of p53 activation, we also evaluated the expression of NAD-dependent deacetylase sirtuin-1 (SIRT1), known to inhibit p53 activity through its deacetylation in Lys 382 (Vaziri et al. 2001), and known to be in turn downregulated by p53 activation (Yamakuchi and Lowenstein 2009). As expected, in accord with p53 acetylation status in HepG2, wt p53 cell line, SIRT1 was slightly down regulated only after 24 h of FFAs treatment (Fig. 3a, c). On the contrary, FFAs-treated Huh 7.5.1 showed an opposite behavior, characterized by an appreciable increase of both SIRT1 transcript and protein at 24 h (Fig. 3b, d).

When we evaluated p21 protein expression, a well-known target of p53, its behavior, as predictable, followed the activity status of wt p53 and Y220C mutated form (Fig. 3a–d). Conversely, when we looked at the expression level of SCO2, involved in copper cellular efflux and cell respiration, it did not reflect the timing of activation of the two different forms of p53, but it was inversely modulated respect to the activity of the two different studied forms of p53 (Fig. 3a–d).

### Copper homeostasis is modulated by p53 response to FFAs treatment

**Fig. 4:**
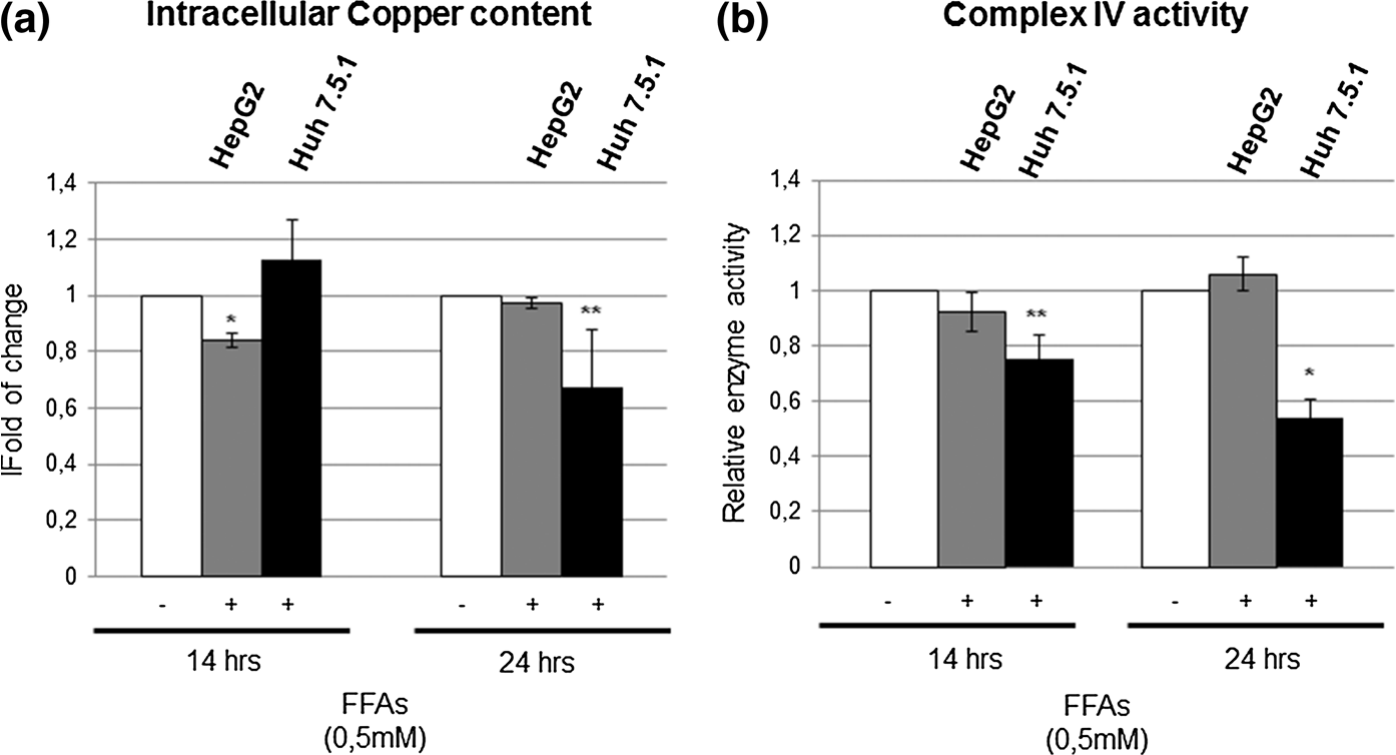
FFAs treatment affects intracellular copper content and Cytochrome *c* oxidase (Cytox) activity in the two cell lines differently. **a** Copper content was evaluated through atomic absorption spectroscopy in whole cells extracts after digestion by HNO_3_. The experiment was repeated three times and the data shown are presented as fold of change versus control ±SD. *p < 0.05; **p < 0.01; ***p < 0.001. **b** Cytox activity was measured in fresh whole cell homogenate by a spectrophotometric assay, by following the oxidation of reduced cytochrome c at 550 nm. The experiment was repeated at least three times, and the data shown are the mean ± SD. *p < 0.05; **p < 0.01; ***p < 0.001

We were intrigued by the unexpected modulation of SCO2, thus in order to understand the biological effects of its deregulation, we evaluated the copper content in the two hepatoma cell lines, after treatment with FFAs. To this aim, we performed atomic absorption spectrometry on cell extracts. Indeed, the intracellular copper modulation well correlated with the modulation of SCO2 protein (Fig. 4a), that, as described before, is able to modulate copper efflux. In HepG2 cells we appreciated, in the first 14 h, a decrease (about 20 %) of intracellular copper content; whereas in Huh 7.5.1 cells, we evaluated an important intracellular copper content reduction (about 30 %), but only after 24 h of FFAs treatment (Fig. 4a). Then, we analyzed the activity of Cytox, which is a copper-dependent enzyme crucial for aerobic respiration. As shown in Fig. 4b, in HepG2 cells FFAs treatment did not significantly affect the Cytox activity. In fact, we appreciated only a slight reduction (10 %) of its activity after 14 h of treatment (Fig. 4b). Conversely, in Huh 7.5.1 cells, after FFAs treatment we appreciated a gradual Cytox enzyme activity reduction, specifically from 20 %, at 14 h, to 40 % at 24 h (Fig. 4b).

In order to understand the mechanism(s) underlying the observed intracellular copper deregulation, we analyzed the expression levels of some key players of copper intracellular homeostasis, such as the copper transporter 1 (CTR1), crucial for copper cell entry, and the ATPases, Cu^2+^ transporting, alpha and beta polypeptides (ATP7A and B), necessary for copper cellular efflux.

**Fig. 5:**
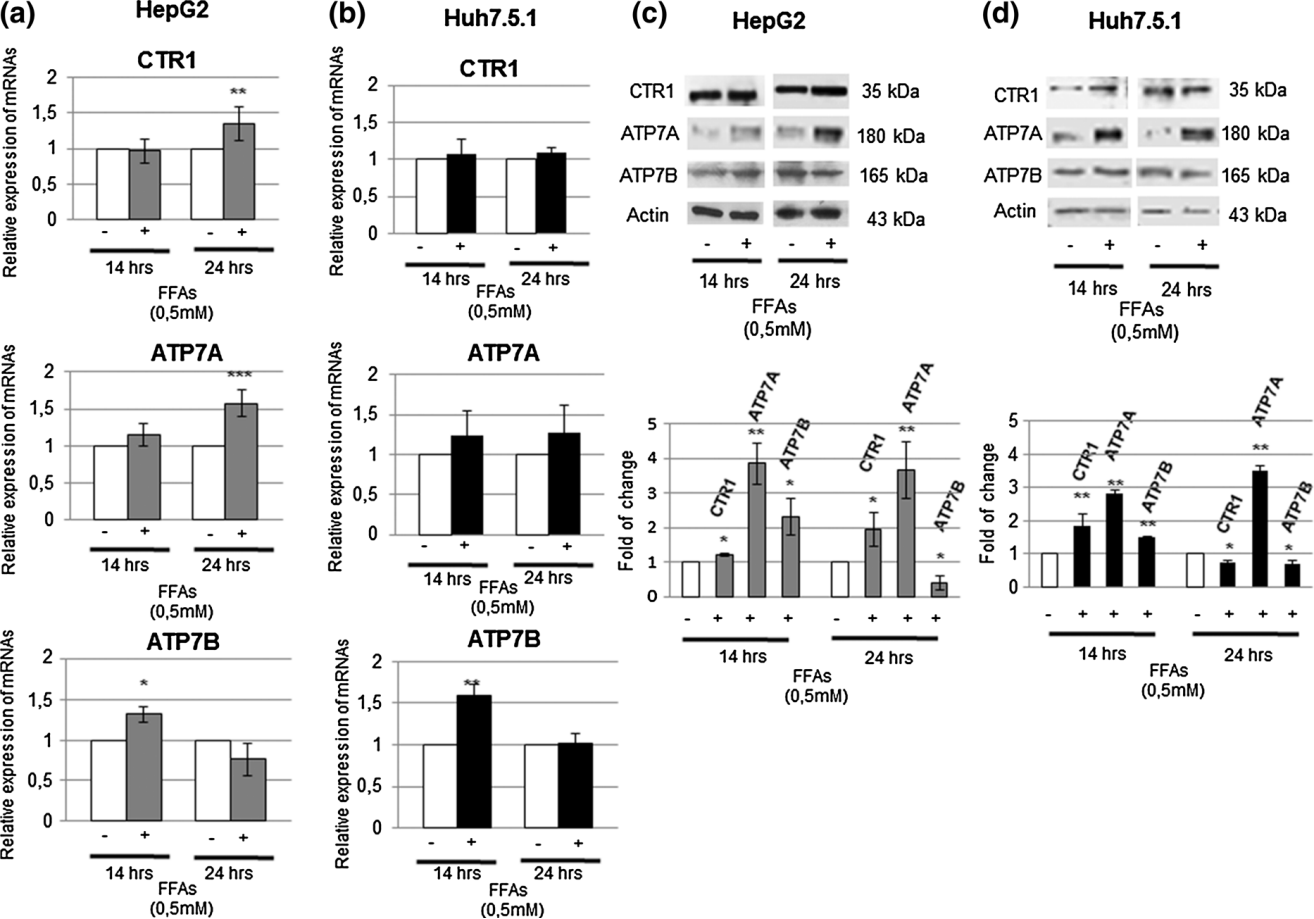
Y220C p53 mutant causes a different modulation of key players in the maintenance of intracellular copper homeostasis in Huh7.5.1 cells FFAs treatment. **a** and **b** Effect of FFAs on CTR1, ATP7A and ATP7B gene expression. mRNAs relative expression was evaluated by RT-PCR in HepG2 and Huh 7.5.1 after 14 and 24 h of treatment; β-actin was used as housekeeping gene. All *data* reported were the mean of three different experiments ±SD. *p < 0.05; **p < 0.01; ***p < 0.001. **c** and **d** The corresponding protein amounts were evaluated by Western Blotting (with relative densitometric analysis) in both HepG2 and HuH7.5.1; β-actin was used as a loading control. *Data* showed are representative of three independent experiments and are expressed as fold of change versus controls ±SD. *p < 0.05; **p < 0.01; ***p < 0.001

Interestingly, in HepG2 cells, CTR1 mRNA evaluation revealed an upregulation at 24 h of treatment (Fig. 5a), on the contrary the protein amount showed a slight but statistically relevant increase of protein amount already at 14 h and up to 24 h (Fig. 5c). In Huh 7.5.1 cells, instead, CTR1 transcript was not affected by the FFAs treatment (Fig. 5b), whereas, the CTR1 protein resulted already significantly up-regulated after 14 h of treatment, and decreased after 24 h (Fig. 5d).

At the same time, we appreciated an interesting modulation of the ATP7A, which showed a marked up-regulation of mRNA expression in HepG2, after 24 h of FFAs treatment (Fig. 5a); however the protein content modulation showed a similar behavior in both HepG2 and Huh 7.5.1 cell lines, with a marked upregulation after 14 h yet (Fig. 5c, d). ATP7B mRNA and protein levels modulation, instead, showed a trend similar in both cell lines (Fig. 5).

### Y220C mutation of p53 protein has a key role in the modulation of copper intracellular homeostasis after FFAs treatment

**Fig. 6:**
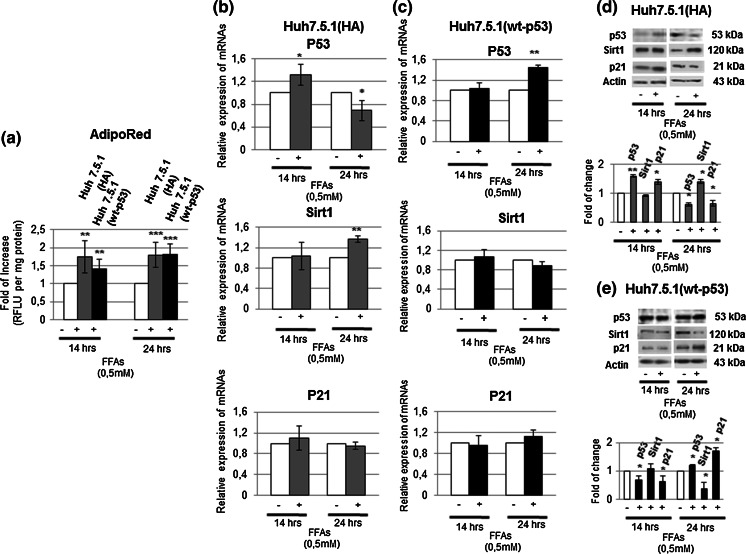
The wt-p53 transfection inHuh7.5.1 cells causes a similar modulation of lipid accumulation, p53, SIRT1 and p21 of those observed in HepG2 naïve cells after FFAs treatment. **a** Lipid accumulation evaluated through Adipored assay in transfected Huh7.5.1 cells. Four different experiments were performed. *Data* are presented as fold of increase of Relative Fluorescence Units (RFLU) per mg of protein compared to controls and are expressed as mean ± SD. *p < 0.05; **p < 0.01; ***p < 0.001. **b** and **c** RT-PCR analysis of p53, Sirt1 and p21 in HA-Huh7.5.1 and wt-p53-Huh7.5.1, respectively; β-actin was used as housekeeping gene. *Data* are representative of four independent experiments ± SD. *p < 0.05; **p < 0.01; ***p < 0.001. **d** and **e** Corresponding proteins expression was evaluated by Western Blotting (with relative densitometric analysis) in HA-Huh7.5.1 and wt-p53-Huh7.5.1, respectively; β-actin was used as a loading control. *Data* are representative of four independent experiments and are presented as fold of change compared to controls ±SD. *p < 0.05; **p < 0.01; ***p < 0.001

In order to ascertain if the Y220C mutation of p53 had a direct role in the copper modulation observed after FFAs treatment, Huh7.5.1 cells were stable transfected with a vector containing the wild-type p53 (wt-p53-Huh7.5.1). Wt-p53- and HA-Huh7.5.1 (those transfected with the empty vector control) cells were treated with FFAs for 14 and 24 h. The presence of the wt-p53 allowed a more gradual intracellular lipid content increase and restored the same molecular response to FFAs treatment observed in HepG2 regarding p53 (Fig. 6a–e).

**Fig. 7:**
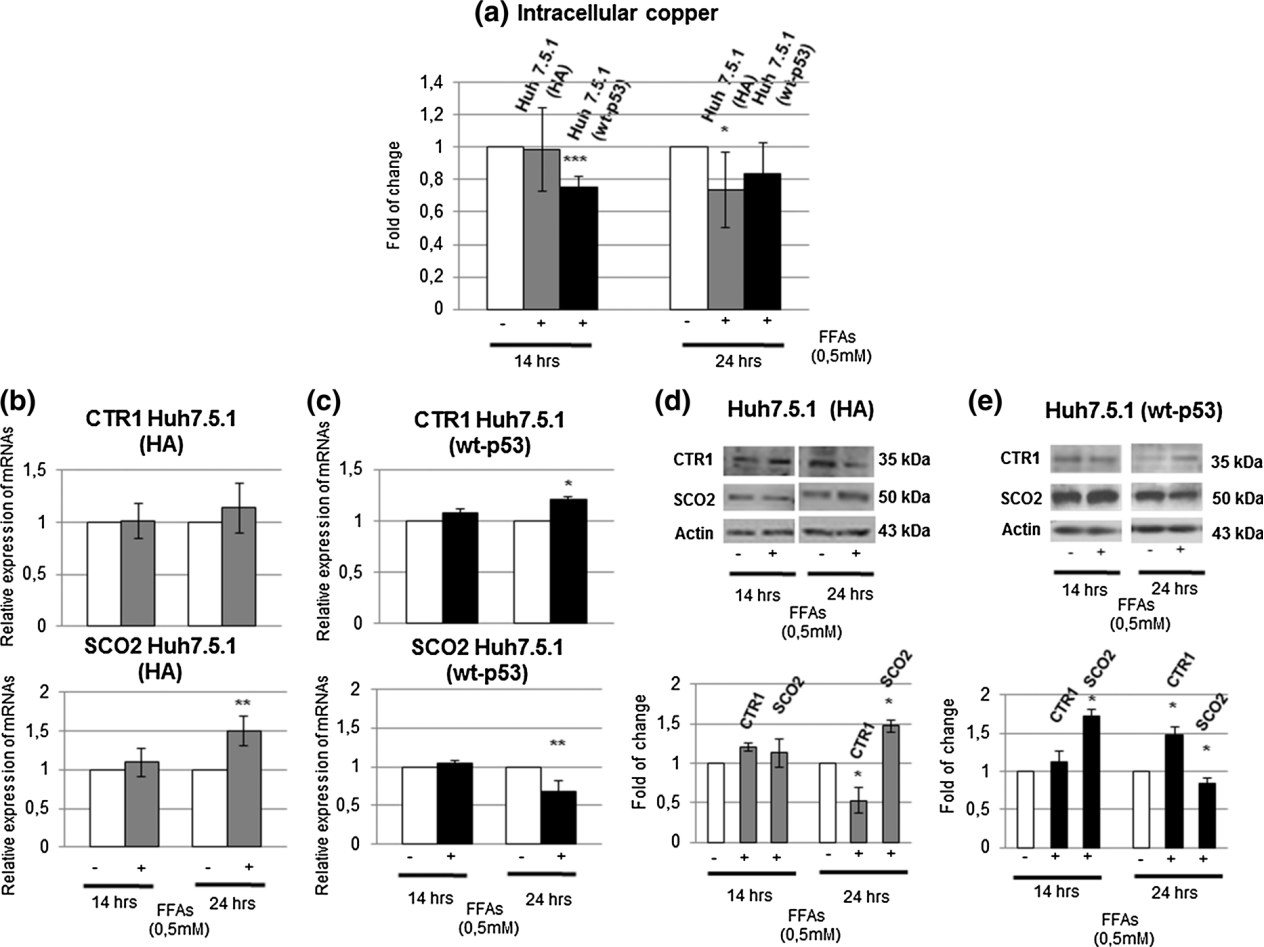
wt-p53 transfection in Huh 7.5.1 causes a modulation of intracellular copper content, CTR1 and SCO2 similar to HepG2 cells in response to FFAs treatment. **a** Copper content was assayed through atomic absorption spectroscopy in whole cell lysates after digestion by nitric acid. The experiment was repeated three times. *Data* shown are expressed as fold of change compared to controls and are expressed as mean ± SD. *p < 0.05; **p < 0.01; ***p < 0.001. **b** and **c** mRNAs expression of CTR1 and SCO2 by RT-PCR analysis in HA-Huh7.5.1 and wt-p53 Huh 7.5.1, respectively; β-actin was used as housekeeping gene. Data are representative of four independent experiments and are expressed as mean ± SD. *p < 0.05; **p < 0.01; ***p < 0.001. **d** and **e** CTR1 and SCO2 proteins expression was evaluated by Western Blotting (with relative densitometric analysis) in HA-Huh7.5.1 and wt-p53-Huh 7.5.1, respectively; β-actin was used as a loading control. *Data* are representative of four independent experiments and are expressed as fold of change compared to the controls ± SD. *p < 0.05; **p < 0.01; ***p < 0.001

When we analyzed, in wt-p53-Huh7.5.1, the intracellular copper content by atomic absorption spectrometry, and the expression pattern of CTR1 and SCO2, the behavior of cells were really close to that observed in HepG2 cells. Cells displayed a decrease of copper intracellular content in wt-p53-Huh7.5.1 of about 20 % after 14 h of treatment, and a slight increase of it after 24 h (Fig. 7a). Accordingly, we highlighted an increase of CTR1 protein expression after 24 h (Fig. 7c, e). In parallel, we appreciated an increase of SCO2 protein content, after 14 h, and a reduction of both mRNA and protein levels, after 24 h (Fig. 7c, e).

Transfection of the wt-p53 did not affect the modulation of ATP7A and B expression patterns by FFAs (data not shown).

## Discussion

NAFLD is a global health burden and a plethora of scientists work every day to understand the molecular basis of its pathogenesis. Unfortunately, despite such huge amount of efforts, the exact mechanism of its pathogenesis is still poorly understood. The discovery that molecules implied in mechanisms like cell death and/or cell cycle control are involved in metabolic regulation, like p53, further widens the fields of investigation to understand the molecular basis of metabolic changes, and indicates how far we are from understanding them.

We have previously demonstrated that the Y220C p53 mutant, one of the most common p53 mutation found in human cancers (Petitjean et al. 2007), in a condition of free fatty acids overload, has a deep impact on cellular lipid metabolism modulation (Gori et al. 2014).

On these bases, using the same cell lines, here we investigated whether p53 differences could have further implications in NAFLD pathogenesis. p53 is known to regulate cellular energy metabolism through the modulation of aerobic respiration and glycolysis; thus, we focused our interest on the regulation of copper homeostasis, important for aerobic respiration. Copper serum levels, in fact, are known to be altered in NAFLD and metabolic syndrome patients (Aigner et al. 2010; Medici 2013). In particular, these patients show reduced hepatic copper content, which is in turn linked, to lipid metabolism alteration and insulin resistance (Medici 2013).

The up-regulation of p53 in response to intracellular copper decrease is not surprising; indeed, in neuroblastoma cells, copper level depletion, produced by a prolonged exposure to a specific copper chelating agent, increased p53 levels and activity (Lombardo et al. 2003).

Here, we highlighted that p53 is involved in the modulation of the intracellular copper homeostasis, in hepatoma cell lines, in response to an excessive fat intake through two distinct actions: negatively, acting on SCO2, thus affecting copper excretion, and promoting the up-regulation of CTR1, thus prompting the increase of cellular copper uptake.

What we revealed is a new p53 function: the copper pool maintenance. The occurrence of copper deregulation, in fact, may be detrimental for several cellular functions, like antioxidant defense and aerobic respiration (Scheiber et al. 2013), and it was proposed to participate in pathogenic mechanisms of NAFLD (Aigner et al. 2010). Interestingly, we appreciated how the activation of both p53 forms (wt and Y220C) has, as consequence, the maintenance of the intracellular copper pool; but they showed marked differences in such modulation. Fatty acids treatment in HepG2 cells, in fact, causes a reduction of copper content, that in turn promotes, at 24 h, the activation of wt p53, maintaining, in this way, the metal content very close to that of control cells. In Huh 7.5.1, instead, the activation of Y220C p53 happens earlier than wt p53, but at prolonged time (24 h) of treatment, the mutated p53 activity decreases, causing a marked reduction of the intracellular copper content.

Thus, we highlighted how the Y220C mutation affects the ability of p53 in controlling the intracellular copper homeostasis (Fig. 4). In fact, while the HepG2 cells are characterized by only a 20 % of intracellular copper content decrease after FFAs treatment, the copper level decreased of about 50 % between 14 and 24 h of treatment in the Huh 7.5.1. In our system, p53 response and the modulation of copper level correlate with Cytox activity. In HepG2 cells, Cytox activity showed a slight reduction after 14 h of FFAs treatment, but at 24 h was fully restored. On the contrary, it resulted gradually and significantly reduced in Huh7.5.1 cells, as well as the intracellular copper content. The impairment of intracellular copper content, in presence of fatty acids excess, and the consequent down-regulation of the aerobic respiration and the ATP production may be deleterious for liver functions and surely may exacerbate NAFLD progression, through the occurrence of a pro-oxidant status and the lack of a an adequate power supply. Copper deficiency, in fact, was associated to mitochondrial dysfunctions, oxidative stress occurrence (Lombardo et al. 2003) and altered lipid metabolism (Medici 2013), all mechanisms also involved in NAFLD pathogenesis (Gambino et al. 2011).

In the light of the data obtained, our investigations revealed that p53 core domain, which carries the Y220C mutation, is involved in the CTR1 and SCO2 expression and activities, in presence of FFAs excess; thus, it is needed for copper intracellular homeostasis.

On the other hand, Y220C p53 has any effects on the two copper pumps responsible of the cellular copper efflux, the ATP7A and ATP7B. The latters, in fact, are similarly modulated in both cell lines, independently by the time of treatment.

The up-regulation of ATP7A that we revealed in these hepatoma cell lines is intriguing. In literature, in fact, is reported that ATP7B is the main copper pump responsible of the efflux of the metal from hepatocytes, while ATP7A is mainly expressed in the other body districts (La Fontaine et al. 2010). Here, we highlight a modulation of this protein in response to an excessive fat intake that far as we know, was not known until today. Thus, under specific conditions, such as metabolic stress, the ATP7A could be overexpressed also at hepatic level, showing that the network at the basis of copper homeostasis maintenance, could be modulated in different ways from those known till today. To note, our investigation also reveals that the up-regulation of ATP7A is independent by p53 expression and activity, but is directly driven by fatty acids accumulation.

We demonstrated, for the first time, that p53 domain carrying the Y220C mutation is involved in copper homeostasis modulation/preservation. For sure, our investigations should be extended to other p53 mutants to understand if other p53 protein domains are involved in copper intracellular homeostasis. This new activity of p53 that we revealed can be considered protective, because it aims to avoid copper homeostasis alteration. In addition, it may have deep implications in other clinical conditions where copper homeostasis is known to be deregulated, such as: aging, cognitive impairment, Alzheimer’s disease, metabolic disorders and cancer.
